# Anchored but not internalized: shape dependent endocytosis of nanodiamond

**DOI:** 10.1038/srep46462

**Published:** 2017-04-13

**Authors:** Bokai Zhang, Xi Feng, Hang Yin, Zhenpeng Ge, Yanhuan Wang, Zhiqin Chu, Helena Raabova, Jan Vavra, Petr Cigler, Renbao Liu, Yi Wang, Quan Li

**Affiliations:** 1 Department of Physics, The Chinese University of Hong Kong, Shatin, New Territory, Hong Kong; 2 Institute of Organic Chemistry and Biochemistry AS CR, v.v.i., Flemingovo nam. 2, 166 10 Prague 6, Czech Republic; 3 University of Chemistry and Technology Prague, Technicka 5, 166 28 Prague 6, Czech Republic; 4 The Chinese University of Hong Kong, Shenzhen Research Institute, Shenzhen, China

## Abstract

Nanoparticle-cell interactions begin with the cellular uptake of the nanoparticles, a process that eventually determines their cellular fate. In the present work, we show that the morphological features of nanodiamonds (NDs) affect both the anchoring and internalization stages of their endocytosis. While a prickly ND (with sharp edges/corners) has no trouble of anchoring onto the plasma membrane, it suffers from difficult internalization afterwards. In comparison, the internalization of a round ND (obtained by selective etching of the prickly ND) is not limited by its lower anchoring amount and presents a much higher endocytosis amount. Molecular dynamics simulation and continuum modelling results suggest that the observed difference in the anchoring of round and prickly NDs likely results from the reduced contact surface area with the cell membrane of the former, while the energy penalty associated with membrane curvature generation, which is lower for a round ND, may explain its higher probability of the subsequent internalization.

Endocytosis of nanoparticles (NPs) serves as the very first step in nanoparticle-cell interactions, the understanding of which is critical in designing NPs for applications such as intracellular sensing and drug delivery. Although this has been an area of focused investigation, the understanding of how the material parameters of the NPs (such as size, chemistry and shape etc.) affect the endocytic process is far from complete.

It is known that endocytosis is an energy dependent process. It has two stages—anchoring followed by internalization. The anchoring process refers to the attachment of NPs to the plasma membrane after numerous random collisions during their Brownian motion in the culture medium. Once NPs are anchored, their engulfing relies on deformation of the cell membrane and is an energy dependent process. Unfortunately, the majority of previous studies did not differentiate these two stages when discussing the NPs’ endocytosis process, although such differentiation could elucidate the correlation between the NP material parameters and the rate-limiting step of endocytosis.

Previous studies showed that the size and surface properties (chemistry and charge) of NPs affected specific endocytosis pathways^1^,^2^, which further determined the intracellular trafficking of the NPs after their cellular entry^3^. The most favourable size range (~50–100 nm, varied in different NP systems) for NP endocytosis was identified, and the energy penalty of plasma membrane deformation was proposed to explain the less favourable endocytosis beyond this range^1^,^4^. Surface properties of the NPs, including both the presence of specific surface chemical species (e.g. ligand, sugar, and protein etc.) and surface charges, were also found to affect the endocytosis process. Specific surface ligand^5^,^6^ as well as positively charged surfaces^3^,^6^,^7^ would enhance the interaction between the NPs and the plasma membrane, and thus increase the probability of endocytosis.

Less is known about the effect of NP’s morphological features on their endocytosis, and the observation was quite scattered^8^,^9^,^10^,^11^,^12^. Some work found nanoparticles with large aspect ratio associated with relatively low cellular uptake, for example, the cellular uptake of Au nanorods was shown to decrease as the rods’ aspect ratio increased^10^. It was also reported that phagocytosis of oblate ellipsoidal polymeric particles were much more significant than that of prolate ellipsoids or spheres^11^. However in another work, It was shown that polystyrene rods exhibited lower nonspecific uptake when compared to their spherical counterparts^12^. The easier cellular entry of particles of certain shapes was generally attributed to their higher anchoring probability and/or easier plasma membrane wrapping based on simulation works^9^.

In the present work, we focus on the endocytosis of nanodiamond (ND), which is an important nanomaterial with wide applications for drug delivery^13^,^14^, bio-tracking^15^,^16^,^17^,^18^, and sensing^18^,^19^,^20^,^21^,^22^,^23^,^24^. show that different morphological features of NDs affected their endocytosis process. Specifically, we identify that the prickly ND has a high anchoring probability but suffers from difficulty internalizing afterwards. In comparison, the anchoring of a round ND (obtained by selective etching of the prickly ND) with moderate probability was followed by easy internalization, leading to a much higher level of endocytosis than its prickly counterpart. Using molecular dynamics (MD) simulations and continuum modelling, we provide qualitative explanations of our experimental findings. Specifically, we show that while the reduced surface areas of round NDs could decrease their anchoring probability compared with the prickly NDs, the energetic cost of membrane wrapping is lower for the round NDs, which, in turn, could provide them with a higher internalization probability than their prickly counterparts.

## Results and Discussions

**Figure 1:**
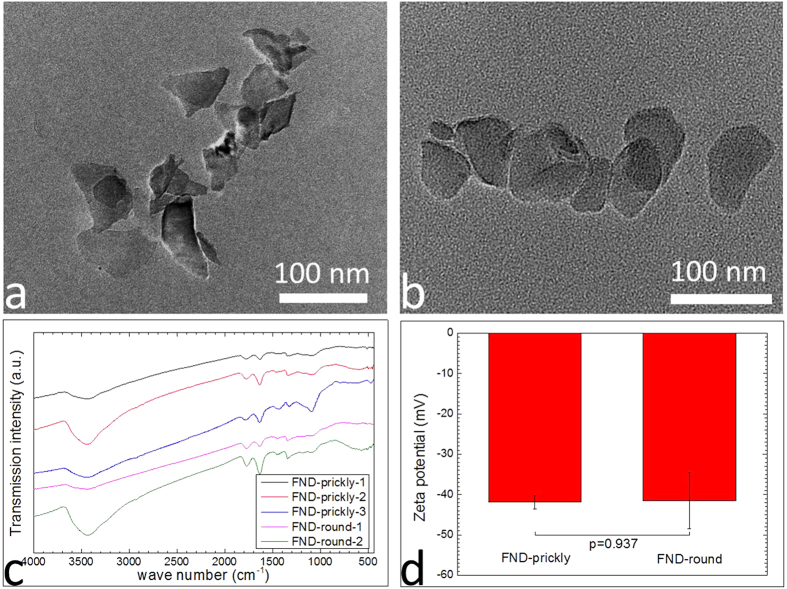
Characterization of prickly and round NDs. TEM images of (**a**) prickly NDs and (**b**) round NDs. (**c**) FTIR spectra of prickly and round NDs, showing that they had similar surface functional group. (Peak intensity of each individual functional group varies from one sample to another. (Peaks at 3400 and 1630 were O-H bending of carboxylic acid, peak at 1780 was belonged to bond of carboxylic acid, peaks between 1450–100 were belonged to the ether like groups). (**d**) Zeta potential of prickly and round NDs (−41.9033 ± 1.6671 for prickly NDs and −41.5567 ± 6.93695 for round NDs. Error bar represents SD, p = 0.937, indicating that there was no significant difference in zeta potential between the two NDs, n = 3).

Both the prickly and round NDs had irregular shapes with size ranging from 30–100 nm, as revealed by the TEM images shown in Fig. 1a,b, and S1. The surface termination of both ND types was normalized after the etching using oxidative mixture of mineral acids. FTIR results (Fig. 1c) taken from the respective samples suggested the presence of similar surface functional groups, i.e., rich in −OH and –COOH, on both prickly and round NDs. The surfaces of both prickly and round NDs were negatively charged with zeta potential of ~−42 mV (Fig. 1d). Both types of NDs have photoluminescence, which was employed to quantitatively identify the amount of ND in the following experiments (Figure S2 & S3). The only feature that differentiated the prickly NDs from the round ones was their morphological characteristics, i.e., sharp edges and corners were always observed in the prickly NDs (Fig. 1a, S1a and S1c), which were absent in the round NDs (Fig. 1b, S1b and S1d).

Upon incubating with HepG2 cells, both the prickly and round NDs were found to take endocytosis as the cellular uptake route, with micropinocytosis as the major pathway (Figure S4). In the first stage of endocytosis, NDs in the medium will anchor to plasma membrane before their internalization by cells. The NDs anchored on plasma membrane showed significantly different behaviour compared with those in the medium and in cytoplasm, as can be clearly seen from the instant velocity and caging diameter (Figure S5).

**Figure 2:**
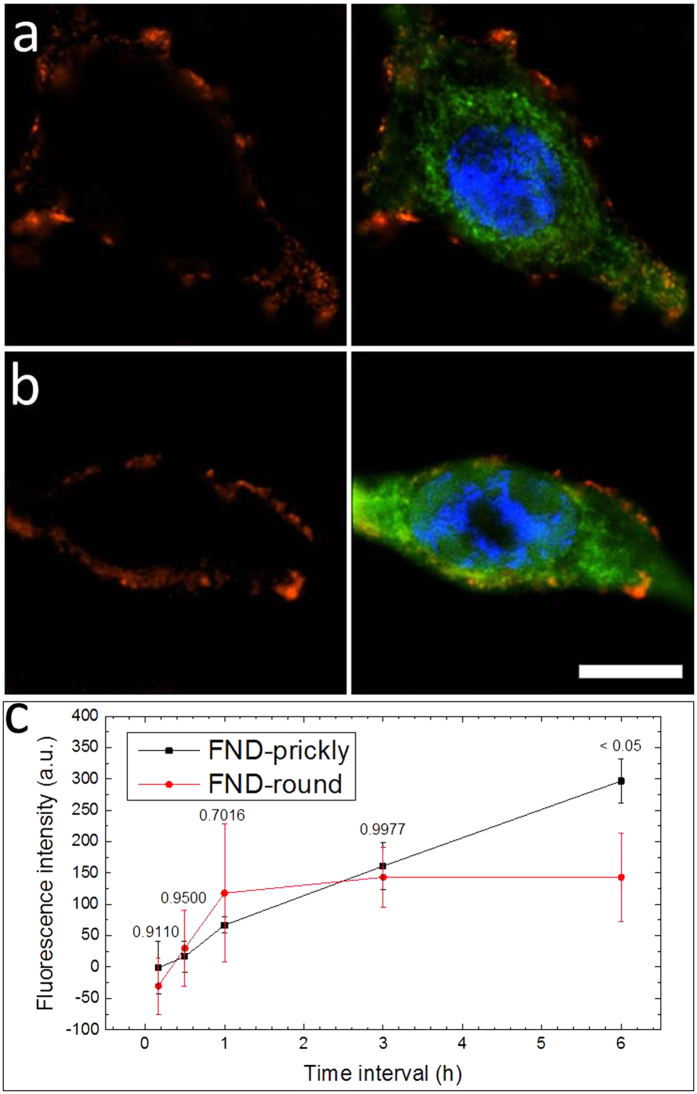
N-SIM images of prickly (**a**) and round NDs (**b**) respectively incubated with HepG2 cells at 4 °C for 6 hrs. (Green: stained cytoplasm; blue: nucleus; red: fluorescence signal of ND; Scale bar is 20 μm). (**c**) Time dependent quantitative results comparing the amount of surface anchored prickly and round NDs (Error bar represents SD. Inserted numbers are p values for the comparison at each time point, n = 4).

To differentiate the anchoring from the internalization process, we carried out the ND-cell incubation at 4 °C. At this temperature, anchoring could take place, while the energy dependent internalization process was largely prohibited. Figure 2a and b compare the anchoring of prickly and round NDs after their incubation with cells for 6 hrs. at 4 °C. Both prickly and round NDs were found to locate on the plasma membrane of the cell and little internalization of NDs was identified in both cases. Anchoring was found to be a time dependent process for both prickly and round NDs. Figure S6 and S7 respectively show and the corresponding N-SIM images of cells incubated with prickly and round NDs for 10 min, 30 min, 1 hr, 3 hrs and 6 hrs. At short incubation durations, the attachment of both prickly and round NDs was limited, and their amount gradually increased as the incubation duration extended. Quantitative results (Fig. 2c) showing time dependent anchoring process of both types of NDs were obtained by measuring the fluorescence intensity of the anchored NDs (See experimental section). The quantitative comparison enabled the identification of subtle difference between cells incubated with prickly or round NDs. Although the anchoring amount of both types of NDs were low when incubation duration was short (up to 3 hrs.), further incubation (to 6 hrs.) showed that the anchoring amount of the prickly NDs was twice of that of the round ones. Similar results were also obtained from Hela cells under similar experimental conditions (Figure S10a).

**Figure 3:**
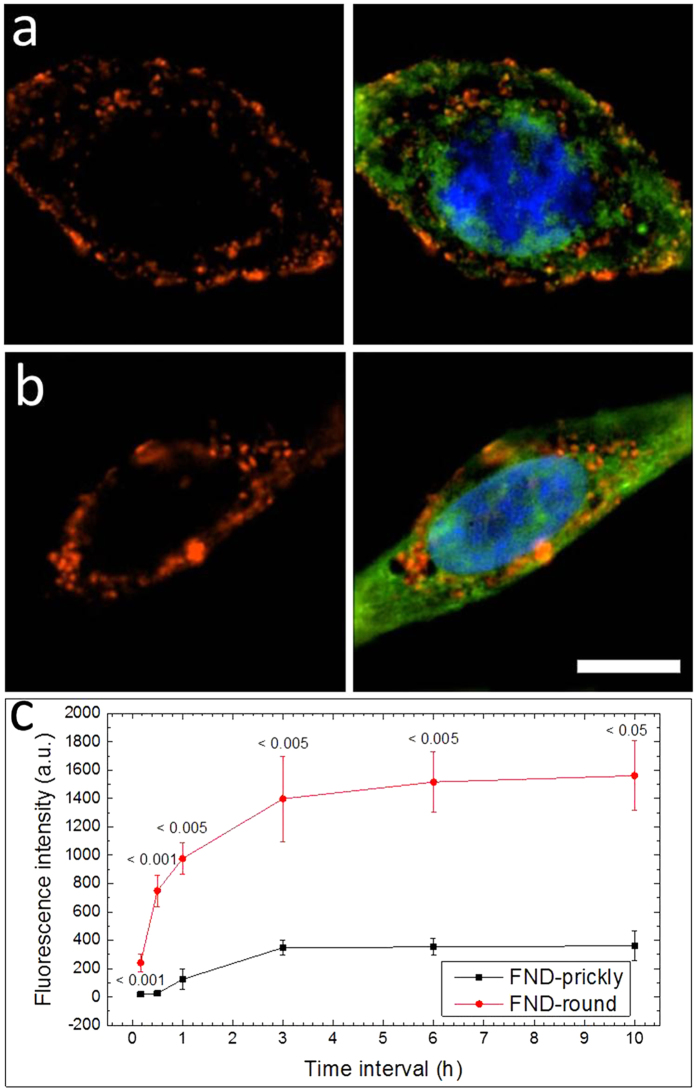
Cellular uptake of prickly NDs and round NDs at normal physiological conditions. Typical N-SIM images of prickly (**a**) and round NDs (**b**) incubated with HepG2 cells at 37 °C for 10 hrs. (Green: stained cytoplasm; blue: nucleus; red: fluorescence signal of ND; Scale bar is 20 μm). (**c**) Time dependent quantitative results comparing the total amount (including both surface anchored and internalized) of prickly and round NDs, the amount of the internalized round NDs was ~4 times higher than that of the prickly NDs (Error bar represents SD. Inserted numbers are p values for the comparison at each time point, n = 4).

To enable ND internalization, the cells were incubated with prickly or round ND at 37 °C. Figure 3a and b compare the cellular uptake of the prickly and round NDs when they were incubated with cells for 10 hrs. Both prickly and round NDs were found inside the cells as well as attached to the plasma membrane. Nevertheless, the majority of the round NDs were found to locate inside the cells rather than anchoring on the plasma membrane. Nevertheless, the majority of the round NDs were found to locate inside the cells rather than anchoring on the plasma membrane. In contrast, the fractions of surface anchored NDs were significant in the case of prickly NDs. The internalization of both types of NDs was also dependent on the incubation duration. Although the N-SIM microscopy provides only semi-quantitative results, a trend of gradually increased ND amount (counting both the internalized and the surface anchored ones) was observed when the incubation duration elongated from 10 min to 10 hrs. (Figure S8 & S9). Quantitative results (Fig. 3c) were obtained by measuring the fluorescence intensity of the endocytic NDs (when both the surface-anchored and the internalized NDs were counted). The general trend was consistent with the N-SIM observation. In addition, a clear difference in the internalization amount can be observed from cell samples fed with prickly and round NDs —the amount of the latter NDs was ~4 times higher than that of the former NDs. Similar trends were obtained in the HeLa cells (SI, Figure S10b).

The anchoring of nanoparticles onto the cell surface is driven by a favorable free energy associated with membrane adhesion^25^,^26^,^27^,^28^,^29^. Such an adhesion energy is often approximated as a linear function of the membrane contact area, *i.e.*, the unit adhesion strength (k_ad_), which is defined as the ratio between the free energy of adhesion and the contact area, is often considered to be a constant for a given type of nanoparticle. This promoted us to examine the difference between the membrane contact areas of the two types of NDs investigated here, in order to understand the difference in their anchoring probabilities.

**Figure 4:**
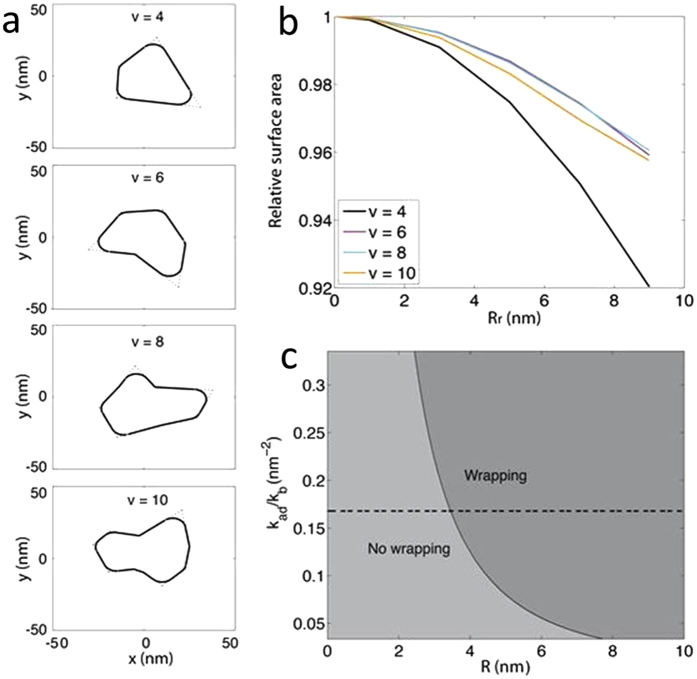
Modelling irregular polygons with rounded corners (**a–b**) and the phase diagram of wrapping the tip of a nanodiamond (**c**). (**a**) Randomly generated irregular polygons with rounded corners. (**b**) The average relative surface area of irregular polygons with 4, 6, 8, or 10 vertices as a function of the rounding radius Rr. The two parameters controlling irregularity (dr and dalpha) are both set to 0.3 (see SI). (**c**) Phase diagram of wrapping a ND tip (wrapping: G_tot_ < 0; no wrapping: G_tot_ > 0). The dashed line indicates the k_b_ = 20 k_B_T and k_ad_ = −2 kcal/(mol nm^2^) case.

The prickly and round NDs were similar in size, geometry (in terms of aspect ratio), and surface properties, with the latter having their corners ‘rounded off’ through chemical etching. If we approximate the ND surface in contact with the membrane as an irregular polygon, the change in its surface area due to rounding can be obtained numerically (see SI). Specifically, for a given number of vertices, we randomly generated 100,000 polygons and computed their surface areas as the rounding radius R_r_ increased. Figure 4a and b clearly shows that the average surface area of the resulting polygons decreases with increasing R_r_. Such an area reduction depends on the rounding radius, the average size and ‘irregularity’ of the polygons. Nonetheless, the trend revealed by Fig. 4 is robust throughout a series of parameter scan (data not shown). This result suggests that the different anchoring probabilities of prickly and round NDs may arise from their different membrane contact areas. However, given the complexity of chemical etching, the computed contact areas should be taken as a qualitative, rather than quantitative estimate of the different anchoring probabilities of the two types of NDs.

Following the anchoring of NDs onto the cell surface, we further probed the energetics of the initial stage of their internalization, a process driven by favorable ND-membrane adhesion and opposed by unfavorable membrane deformation^30^,^31^,^32^,^33^,^34^,^35^. In order to quantify the unit adhesion strength k_ad_ of NDs investigated here, we determined the binding free energy profile, i.e., the potential of mean force (PMF), of two ND slabs with a POPC lipid bilayer via a total of 1080-ns umbrella sampling calculations. Specifically, k_ad_ is obtained through dividing the free energy depth by the ND slab area. The free energy depth for the 2 nm × 2 nm ND slab and the 4 nm × 4 nm ND slab is 9.93 ± 0.6 and 37.39 ± 1.27 kcal/mol, respectively, which yields very similar k_ad_ values (2.48 ± 0.15 and 2.33 ± 0.08 kcal/mol/nm^2^, respectively), indicating that our result is not significantly affected by the size of the ND slab used in the calculation. In order to evaluate the effect of bilayer size, we repeated the calculation using the 4 nm × 4 nm ND slab with a larger lipid bilayer (see Methods). The newly obtained k_ad_ (2.22 ± 0.07 kcal/mol/nm^2^) is again in reasonable agreement with the aforementioned values. While these results indicate that the computed k_ad_ is largely independent of system size, we note that the shape of the free energy profiles is size dependent. Such dependence is further discussed in the supplementary material.

The average value of k_ad_ from the above calculation (2.34 kcal/mol/nm^2^) was used to determine the energetic cost of membrane wrapping around NDs. Here, we consider only the initial stage of membrane wrapping and focus on the tip of a nanodiamond, where the curvature tends to be the largest (sharpest) and the deformation of membrane is energetically the most costly.

Assuming an infinite, tension-less membrane, the total energy of the ND tip-membrane system (G_tot_) is a function of k_ad_, k_b_, which is the bending modulus of the membrane, the wrapping angle θ, and the radius of the ND tip R (See SI)^26^,^32^,^36^. At fixed k_b_ and k_ad_, the energetic cost of membrane wrapping increases as the tip of the ND sharpens. Considering the heterogeneity of cellular membranes and the error in the calculated ND-membrane adhesion strength, we scanned a series of k_b_ and k_ad_ values. The generated phase diagram (Fig. 4c) indicates that as the ratio of k_ad_ to k_b_ decreases, it becomes increasingly difficult to wrap a prickly ND tip, while wrapping of a round ND tip may still be energetically favorable. This result is in line with more advanced modelling of membrane vesicles where both stretching and bending are considered^27^.

Based on the above calculations, we hypothesize that the higher internalization probability of round NDs may be linked to their lower energetic cost of initializing membrane curvature than the prickly NDs. It has been suggested that upon the endocytosis of viruses, their intrinsic curvatures and abilities to directly interact with membrane components may immediately promote membrane curvature^37^. This may induce the accumulation of specific lipids and curvature-sensing proteins, which then synergize to stimulate subsequent endocytosis^38^. Given that the membrane curvature generated by the round ND tips and viruses are both on the order of tens of nanometers, it is conceivable that similar effects may be at play here, *i.e.*, round NDs more readily initiate membrane curvature than prickly NDs, and this generated curvature in turn triggers further membrane deformation that eventually leads to the internalization of the nanodiamonds. Note that the above hypothesis does not rest on the membrane wrapping seamlessly over an entire ND. Rather, it concerns only the initial stage of internalization, *i.e.*, when membrane deformation begins to develop. While this hypothesis remains to be further examined, especially in the context of micropinocytosis, the energetics of membrane wrapping discussed here is in line with previous studies on the endocytosis of nanoparticles with diverse physico-chemical properties^4^,^33^,^39^,^40^,^41^.

## Conclusions

In conclusion, we differentiated the anchoring and internalization stages during NDs’ endocytosis process by carrying out the respective experiments at 4 and 37 °C. We discovered that the morphological features of the NDs, i.e. prickly ones with sharp edges/corners vs. the round ones with sharp edge/corners etched off, were affected at both stages of the endocytosis process. Etching the prickly NDs led to slightly decreased surface area and the disappearance of the sharp edges/corners in the round NDs, these two factors were found to effect (result of) the endocytosis process. Anchoring of both prickly NDs and their round counterparts were observed at 4 °C, with the former showed higher anchoring amount at longer feeding durations (>3 hrs.). This result may be understood as originated from the contact surface area difference of the two types of NDs with the plasma membrane. Interestingly, the prickly NDs did not have to a higher internalization amount, as compared to that of the round NDs. MD simulation and continuum modelling results suggest that the energy penalty associated with membrane curvature generation is lower for a round ND, which may increase its probability of the subsequent internalization. The present study provides general insight to the design of NPs with specific morphological parameters (contact surface area and local sharpness at the interaction point) for controllable cell membrane anchoring and/or endocytosis, which is a critical step for drug delivery and intracellular sensing.

## Methods

### Fabrication and characterization of ND samples

The prickly shape NDs were prepared by oxidation of high-pressure high-temperature (HPHT) NDs (MSY 0–0.05 μm, Microdiamant, Switzerland) in air (510 °C, 5 hrs.) and consequently in a mixture of HF (40%) and HNO_3_ (68%) (2:1 vol.) at 160 °C for 48 hrs. Oxidized NDs were then centrifuged, washed with water, NaOH (1 M), HCl (1 M) and five times with deionized water. Finally, the NDs were concentrated and freeze-dried.

Round NDs were produced by a short-term oxidation of as bought prickly HPHT in molten potassium nitrate 1:200 (w/w)^42^,^43^. Prickly NDs were mixed with one half of KNO_3_ amount and grinded to a fine powder. The other half of KNO_3_ was heated to 575 °C in a vertical tube furnace and under stirring the ND + KNO_3_ mixture was added. After 8.5 min the melt was slowly poured into cold deionized water, KNO_3_ was dissolved and NDs were centrifuged and oxidized in a mixture of HF/HNO_3_ and further processed in the same way as the prickly NDs.

Oxidized prickly and round NDs were consequently irradiated by high-energy protons as described previously^44^, annealed under Ar atmosphere at 900 °C for 1 hr. and oxidized in air and acids as described for prickly NDs in order to normalize their surface chemistry.

The morphology and size of NDs were investigated by TEM (FEI TS12). The surface chemistry of NDs was determined by Fourier Transform Infrared Spectrometer (FTIR, Nicolet 670, Thomas Nicolet, Waltham, MA). The average zeta potential of NDs in water and hydrodynamic diameter of NDs were obtained by DLS (Delsa Nano, BECKMAN COULTER). The fluorescence of NDs in water at concentration of 0, 5, 10, 15 and 20 μg/mL was measured by photoluminescence (Hitachi F7000) with a continuous-wave 535 xenon lamp, the slopes of the linear fit of the emission peak at 761 nm were used to normalize the fluorescence intensity of prickly and round NDs at the same concentration.

### Introducing NDs to cells

Human liver carcinoma HepG2 and cervical carcinoma HeLa cells were used in this study. The cells were cultured with Dulbecco’s modified Egale’s media (Life Technology, HK), supplemented with 10% fetal bovine serum (Life Technology, HK). Cells were grown in a standard cell culture incubator at 37 °C with 5% CO_2_ in a humidified atmosphere. NDs at 20 μg/mL was fixed through the study, cells were fed with NDs at different time interval after being washed with PBS twice.

### N-SIM characterization of cells

Super resolution microscopy (Nikon Super resolution microscope, N-SIM) was used to study the endocytosis of NDs by cells at 37 and 4 °C. For 37 °C, 5 × 10^4^ cells in 2 mL DMEM with 10% FBS were seed on cover glass in a 6 well plate, after 24 hrs incubation, cells were washed with PBS, and fed with NDs in serum free medium. At different time interval, cells were harvested, washed with PBS, fixed with 4% paraformaldehyde at room temperature, stained with Cell Tracker (Invitrogen), and observed by using N-SIM. For 4 °C, it was similar to that of 37 °C with slightly modification. After the 24 hrs. incubation, cells were incubated in 4 °C for 1 hr., washed with ice cold PBS, fed with NDs in ice cold serum free medium, and incubated in 4 °C. The rest procedure was the same as that at 37 °C.

### Quantitative study of endocytosis

Colours in N-SIM images only represented the location of NDs, but not the fluorescence intensity. To quantify the anchored and internalized NDs, a method developed by S. Barua, J.-W. *et al*.^12^ was employed. In this method, 4 °C was used to determine the quantity of anchored particles; and 37 °C was used to let particles both anchor and enter the cells. The quantity of anchored particles was directly related to the fluorescence intensity at 4 °C, and the quantity of internalized particles was calculated by subtracting the fluorescence intensity at 4 °C from that at 37 °C.

In briefly, fluorescence intensity signal of NDs were used for quantitative analysis of endocytosis at 37 and 4 °C. For 37 °C, cells were seeded in black walled 96 well plates at concentration of 5000 cells per well and allowed to grow for 24 hrs. The cells were washed with PBS, fed with NDs in serum free medium. At different time interval, cells were washed with PBS, and kept in 100 μL PBS per well. Fluorescence intensities of the NDs from each well were measured using a microplate reader (Tacan Infinite) at excitation and emission wavelength of 535 and 761 nm, respectively. For 4 °C, after the 24 hrs. incubation at 37 °C, cells were incubated at 4 °C for 1 hr., washed with ice cold PBS, fed with NDs in ice cold serum free medium, and incubated in 4 °C. The rest procedure was the same as that at 37 °C, except ice cold PBS was used through this part.

### Molecular dynamics simulations and continuum modelling

Two nanodiamond slabs (2 nm × 2 nm × 0.5 nm and 4 nm × 4 nm × 0.5 nm) were created following the protocols detailed in our previous work^25^ and briefly summarized in the SI. The ND-membrane systems were then constructed by combining the ND models with a POPC bilayer with 85 lipids in each monolayer and previously equilibrated in a 1-μs simulation^45^. A system with ND slab size of 4 nm × 4 nm × 0.5 nm and 192 lipids in each monolayer, which was previously equilibrated for 50 ns, was constructed to further examine the finite size effect from the membrane.

Prior to umbrella sampling, each of the two ND-membrane systems was minimized for 1000 steps and equilibrated for 10 ns. The reaction coordinate (d) for umbrella sampling was set to the distance between the center-of-mass (COM) of the functional groups on the ND bottom surface and the COM of upper-monolayer phosphorus atoms of POPC projected onto the membrane normal (z-axis, see Fig S11a). Altogether 12 umbrella windows at a spacing of 0.15 nm were generated for the 2 nm × 2 nm × 0.5 nm ND system. In order to achieve improved convergence, an extra window was added for the 4 nm × 4 nm × 0.5 nm ND system and the 4 nm × 4 nm × 0.5 nm ND system with a large bilayer at z = 1.58 nm and z = 1.88 nm, respectively. Each umbrella window was simulated for 30 ns, with a force constant of 400 kcal/mol/nm^2^. Three additional, 30-ns simulations for z = 1.35 nm, z = 1.5 nm and z = 1.58 nm of the 4 nm × 4 nm × 0.5 nm ND system and for z = 1.8 nm, z = 1.88 nm and z = 1.95 nm of the 4 nm × 4 nm × 0.5 nm ND system with a large bilayer were performed, respectively, in order to reduce the relatively large errors for these windows. Data from all umbrella windows were combined with the GROMACS g wham tool^46^. The overlap between adjacent umbrella windows and the convergence of the result is illustrated in Fig S10. In the continuum modelling of the ND tip-membrane system, G_tot_ = G_bend_ + G_ad_ = 4πk_b_(1-cosθ) + 2πR^2^k_ad_(1-cosθ)^26^,^32^, where θ and R denote the wrapping angle and the radius of the ND tip, respectively (Fig S11c).The value of k_ad_ was obtained from the above umbrella sampling calculation, while a typical k_b_ value of 20 k_B_T for lipid bilayers was adopted^26^,^47^. To further probe the energetics of membrane wrapping around a ND tip, a series of k_ad_ and k_b_ values were scanned to generate Fig 4. For additional computational details, please refer to the SI.

## Additional Information

**How to cite this article:** Zhang, B. *et al*. Anchored but not internalized: shape dependent endocytosis of nanodiamond. *Sci. Rep.* **7**, 46462; doi: 10.1038/srep46462 (2017).

**Publisher's note:** Springer Nature remains neutral with regard to jurisdictional claims in published maps and institutional affiliations.

## Supplementary Material

Supplementary Information
