# Supplementary material for: Anchored but not internalized: shape dependent endocytosis of nanodiamond
Source: Sci Rep. 2017 Apr 13;7:46462. doi: 10.1038/srep46462 (PMC5390292; doi:10.1038/srep46462)
Supplement: Supplementary Information [file srep46462-s1.pdf]

Supporting Information

Anchored but not internalized: shape dependent endocytosis of nanodiamond

Bokai Zhang[1], Xi Feng[1], Hang Yin[1], Yanhuan Wang[1], Zhiqin Chu[1], Zhenpeng Ge[1], Helena Raabova[2,3], Jan Vavra[2,3], Petr Cigler[2], Renbao Liu[1,4], Yi Wang[1,*], Quan Li[1,4,*]

[1]Department of Physics, The Chinese University of Hong Kong, Shatin, New Territory, Hong Kong.
[2]Institute of Organic Chemistry and Biochemistry AS CR, v.v.i., Flemingovo nam. 2, 166 10 Prague 6, Czech Republic
[3] University of Chemistry and Technology Prague, Technicka 5, 166 28 Prague 6, Czech Republic
[4] The Chinese University of Hong Kong, Shenzhen Research Institute, Shenzhen, China

[*]yiwang@phy.cuhk.edu.hk; liquan@phy.cuhk.edu.hk

Generation and rounding of irregular polygons

The vertices of a regular polygon are distributed on a circle with a given radius (r); while the angle between the vectors connecting the circle center and any two neighboring vertices is alpha = 2π/v, where v represents the number of vertices. In irregular polygons, both r and alpha can be varied. Here, such variation is controlled through two random numbers, dr and dalpha: a value of 0 indicates no irregularity, while a value of 1 indicates the highest irregularity. To generate models that match NDs studied in the experiments, the average radius r was set to 25 nm. A series of dr and dalpha values were scanned and polygons with up to 10 vertices were investigated.

Model Construction for MD simulations

Following the protocols detailed in our previous work[1], approximately 35% of ND surface carbons were functionalized by hydroxyl (−OH) groups; the ND surface facing a membrane was further functionalized with carboxylate groups (−COO−): 2 and 9 –COO- groups were introduced in the 2nm x 2nm and 4nm x 4nm ND slabs, respectively, creating a charge density similar to those used in our previous work[1]. The remaining carbon atoms on the ND surface were terminated by hydrogen atoms. The POPC bilayer used to construct the two ND-membrane systems contained 85 lipids in each monolayer and was previously equilibrated in a 1-μs simulation performed on the specialized machine Anton[2]. The resulting ND-membrane systems were neutralized by adding sodium and chloride ions at a concentration of 0.1 mol/L. The final ND-membrane systems contain ~55 000 atoms, with a size of approximately 7.5nm x 7.5nm x 9.4nm. A third ND-membrane system was

constructed to evaluate the impact of the size of the bilayer, which contained a 4nm x 4nm ND slab and a bilayer with 192 lipids in each monolayer. This bilayer was previously equibrated for 50 ns.

Umbrella Sampling

Following our previous work[1], a constraint was applied to ensure that the ND slab does not tilt in the xy plane during the umbrella sampling, i.e., the ND surface was kept parallel to the membrane. The initial structures of the umbrella windows were extracted from an equilibrium simulation trajectory. Results of the umbrella sampling calculation were then used to determine $k_{ad} = \Delta G/A_{ad}$, where ΔG is the free energy minimum revealed by the potential-of-mean-force profiles and A_{ad} is the contact area of the ND surface parallel to the membrane.

MD simulation protocols

All simulations were performed with the 2.9 release of NAMD[3], with the cGenFF[4] force field for small molecules and the CHARMM36[5] force field for lipids. TIP3P water model was adopted for all simulations. A time step of 2 fs was adopted in all simulations, with bonds involving hydrogen atoms constrained using RATTLE[6] and water geometries maintained using SETTLE[7]. The multiple-time-stepping algorithm was used, with short-range forces calculated every step and long-range electrostatics calculated every two steps. The cutoff for short-range non-bonded interactions was set to 12 Å, with a switching distance of 10 Å. The CHARMM force-switching scheme was used for vdW force calculation. Assuming periodic boundary conditions, the Particle Mesh Ewald (PME) method[8] with a grid density of at least 1/Å3 was employed for computation of long-range

electrostatic forces. Langevin dynamics with a damping coefficient of 1 ps–1 was used to keep the temperature constant at 310 K, while a Nosé–Hoover–Langevin piston[9] was used to keep the pressure constant at 1 atm. The pressure control was performed semi-isotropically: the z axis of the simulation box, which is normal to the membrane, was allowed to fluctuate independently from the x and y axes.

Continuum modeling

We consider the spherical tip of a nanodiamond in contact with a flat, infinite membrane under zero surface tension (Fig S11). The system's total energy is given by $G_{tot} = G_{bend} + G_{ad}$, with the latter two components representing the bending energy of the membrane and the ND-membrane adhesion energy, respectively:

$$G_{bend} = \int [\frac{1}{2} k_b (c_1 + c_2 - c_0)^2 + \bar{k} c_1 c_2] \ dA$$

$$G_{ad} = k_{ad} * A_{ad}$$

where c_1 and c_2 are the two principle curvatures at a given point on the membrane surface and c_0 is the spontaneous curvature of the membrane (c0=0 here); k_b, $\bar{k}$, and k_{ad} are the membrane bending modulus, saddle-splay modulus, and the ND-membrane adhesion strength, respectively. Based on the Gauss-Bonnet theorem, the term $\int \bar{k} c_1 c_2 \ dA$ yields a topological invariant and makes no contribution to the change in Gbend. Following Deserno[10], we divide the membrane into the part in contact with the nanodiamond (ND-bound part) and the part that is not (free part). Evaluation of Gbend and Gad for the ND-bound part immediately yields $G_{bend} = 4\pi k_b(1-\cos\theta)$ and $G_{ad} = 2\pi R2 k_{ad}(1-\cos\theta)$, where θ and R stand for the wrapping angle and radius of the ND tip, respectively.

The free part of the membrane does not have adhesion energy and the minimal surface property of catenoids can be used to show that its bending energy is exactly zero[10]. Taken together, the total energy of the ND-membrane system is $G_{tot} = 4\pi k_b(1\text{-}\cos\theta) + 2\pi R2k_{ad}(1\text{-}\cos\theta)$. Wrapping of the ND tip is achieved when Gtot is negative, which solely depends on R, k_b and k_{ad} (Fig 4c).

Size dependence of the free energy profiles

As shown in Fig S11, the shape of the free energy profiles depends on the size of the ND slab as well as the bilayer. Specifically, the slopes of the free energy curves change at slightly different locations for the three systems studied here: such a change in slope occurs at z≈1.3, 1.5 and 1.8 nm for the 2 nm × 2 nm ND slab, 4 nm × 4 nm ND slab and 4 nm × 4 nm ND slab with a large bilayer, respectively. As illustrated in Fig S13, these locations correspond to where the ND slab first establishes contact with the lipid membrane. Once such contact is established, the free energy reduces more rapidly (larger slope), reflecting the favorable non-bonded interactions between ND surface and the membrane, as already observed in our previous study (1). Due to its elasticity, a lipid bilayer bends towards the ND slab when the two first establish contact. The former then resumes a flat shape as the ND slab adheres to its surface. Compared with the 2 nm x 2 nm slab, the 4 nm x 4 nm ND slab interacts more strongly with a membrane, thereby, triggering an earlier onset of bilayer conformational change (Fig S13). Furthermore, due to the finite size effect, a larger bilayer is known to be less rigid than a smaller one in simulations (2, 3), allowing the former to bend towards the ND slab at an even earlier location (z=1.8 nm). Despite these differences, it is worth emphasizing that

the final value of the the free energy depth, which measures the affinity of a ND slab adhered to a flat membrane, is largely independent of the slab or bilayer size (Fig S11).

Two-dimensional trajectory of NDs

HepG2 cells were seeded in Petri dishes 24 hrs. before the experiments. NDs were diluted with serum free DMEM to a concentration of 1 μg/mL and added to the Petri dish that washed with PBS for three times. The cells were further incubated for 10 min for observing the anchoring process and 24 hrs for tracking inside the cells. Laser confocal microscope (Leica SP5) was used to track the motion of NDs. The 633nm laser was used for imaging. The transmission light was collected for cell imaging while the scattering light was collected for ND imaging. A video was recorded to track the motion of a single ND, during which the focus was manually adjusted to follow the motion in z-direction. The two dimensional trajectory of ND is extracted by determining its position in each frame with the help of ImageJ software, based on which the instant velocity was calculated. The caging diameter of the trajectories were calculated based on the reported method[11]. A sliding window of 11 frames was set, in which the maximum distance reached was determined and plotted.

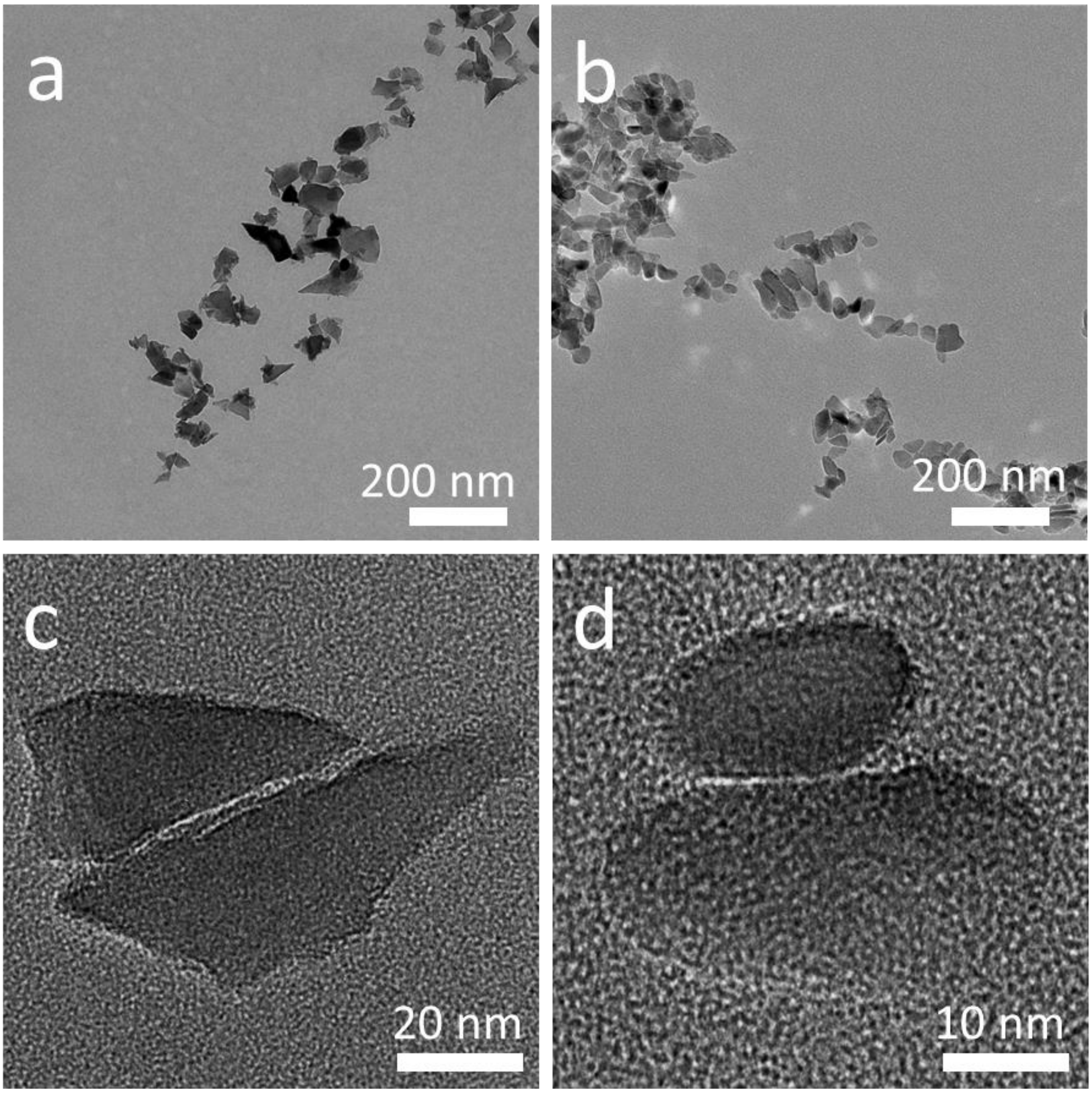

Figure S1. TEM images of (a) & (c) prickly NDs and (b) & (d) round NDs at different magnification.

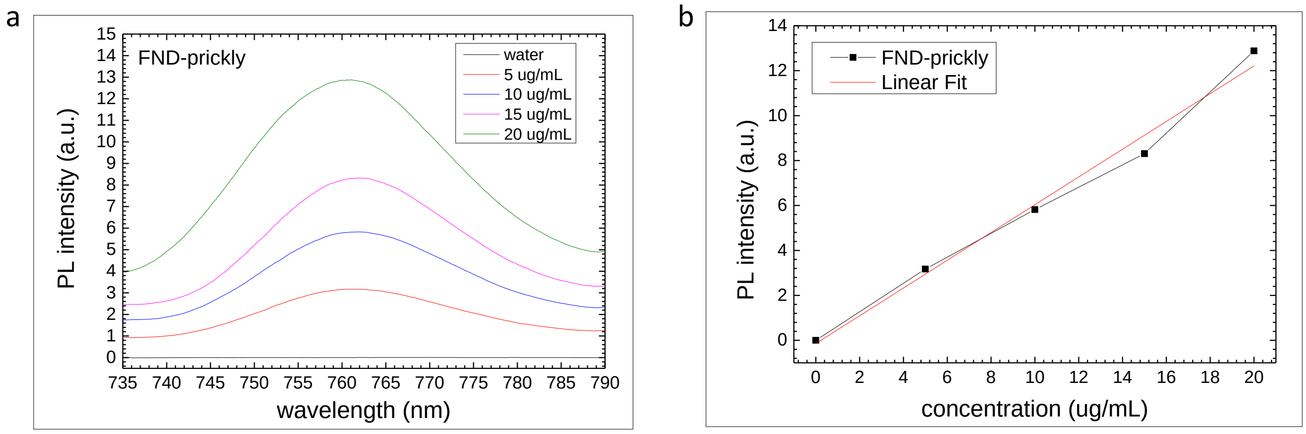

Figure S2. Photoluminescence of prickly NDs used for concentration calibration. (a) PL spectra were taken from ND samples of different concentrations; (b) Fitting of the raw data obtained from (a) showing a linear dependence of PL signal on the ND concentration (Y=(0.6179±0.04052)X+(-0.1438±0.49627), R^2=0.98302).

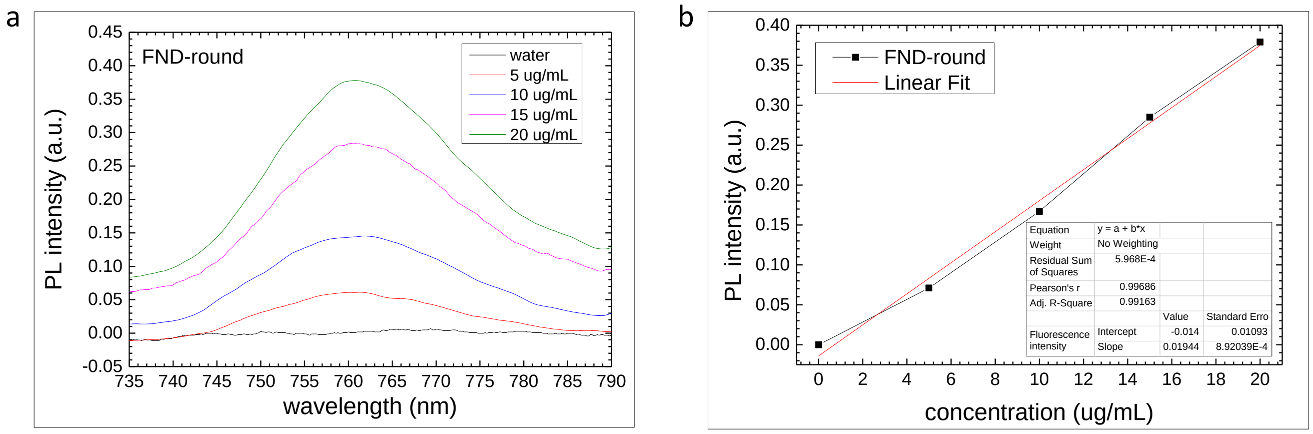

Figure S3. Photoluminescence of round NDs used for concentration calibration. (a) PL spectra were taken from ND samples of different concentrations; (b) Fitting of the raw data obtained from (a) showing a linear dependence of PL signal on the ND concentration (Y=(0.01944±0.000892039)X+(-0.014±0.01093), R^2=0.99163).

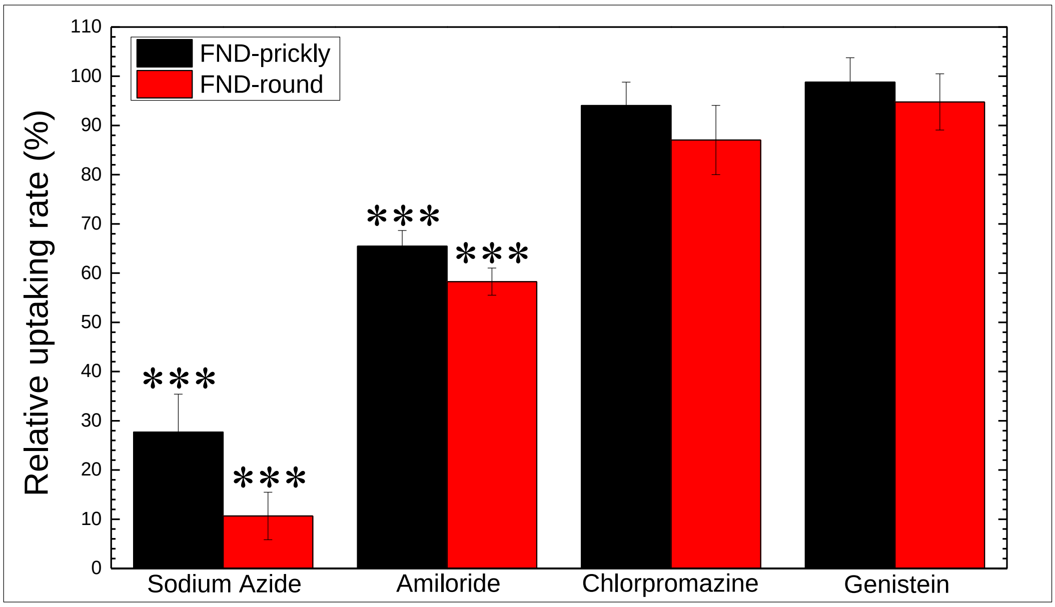

Figure S4. (a) Cellular uptake pathways of NDs assessed by treating the cells with different inhibitors of endocytic routes. Sodium Azide was used as a metabolic inhibitor as it prevents the production of ATP by interfering with glycolytic and oxidative metabolic pathways of cells. Chlorpromazine was used as a specific inhibition of clathrin-mediated endocytosis as it is known to impede the formation and budding of clathrin-coated pits. Genistein perturbs cholesterol-rich membrane microdomains which precursors of caveolae invaginations. Amiloride is an inhibitor of Na+/H+ exchange protein and was used to hinder macropinocytosis. (Briefly, cells were pretreated with varous inbibitors for 1 hr., then NDs (20 µg/mL) were added, followed by another 6 hrs. Incubation. Finally, the cells were washed with PBS, trypsinized, and then processed for flow cytometry. The inhibitor concentrations used were: Sodium azide: 10 mM, Amiloride: 85 µM, Chlorpromazine: 150 µM, Genistein: 185 µM.) (All data were shown as mean ± SD and data of Sodium Azide and Amiloride were significantly different (*** represents $p < 0.05$) from control);

	Name	Events	Cell Death Parent (%)
Control	Control (orange)	494	4.9
FND-prickly	10 mM Sodium Azide (black)	428	4.21
	185 µM Genistein (indigo)	531	4.96
	150 µM Chlorpromazine (gray)	375	3.71
	85 µM Amiloride (blue)	402	4.01
FND-round	10 mM Sodium Azide (black)	382	3.84
	185 µM Genistein (indigo)	367	3.7
	150 µM Chlorpromazine (gray)	427	4.27
	85 µM Amiloride (blue)	402	4.03

Table S1. Cell death percentage in 10000 event for each sample.

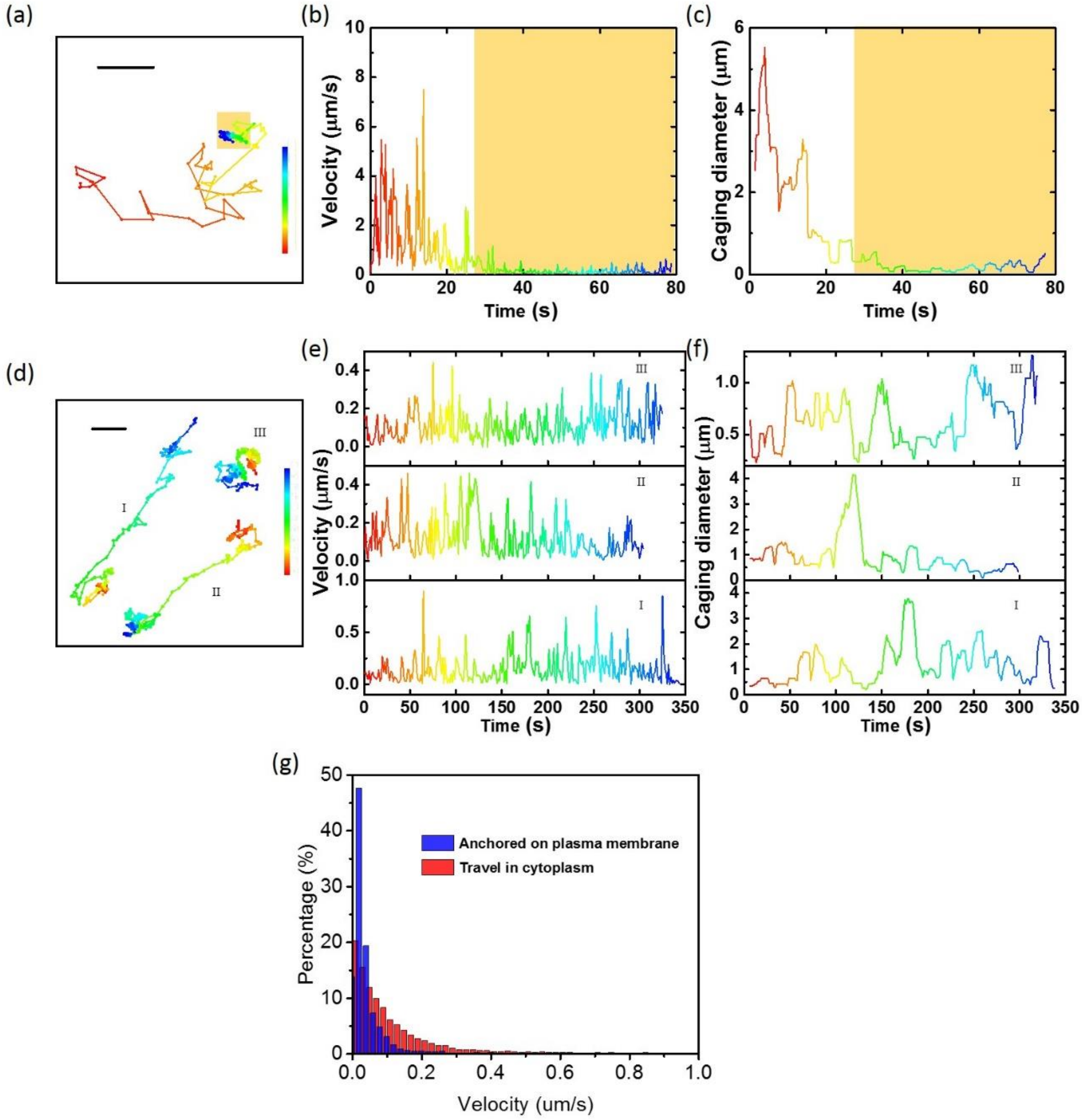

Figure S5. (a) Typical trajectory of prickly shape ND approaching and anchoring onto the plasma membrane, represented by time-coded color map. Red: starting point; Purple: ending point; (b) The corresponding instant velocity of the same ND with trajectory shown in (a); (c) Caging diameter of the same ND with trajectory shown in (a); (d) Three most typical states of NDs in cell after endocytosis: directed motion (I), confined motion (III) and the mixed state (II); (e) The corresponding instant velocity of the same NDs with trajectory shown in (d); (f) Caging diameter of the same NDs with trajectory shown in (d). Scale bars represent 2 μm in both (a) and (c); (g) Distribution of velocities for NDs anchored on plasma membrane and travel in cytoplasm.

In the first stage of endocytosis, both types of NDs were found to anchor on the plasma membrane of the cells. This was clearly demonstrated in Figure S5a, which shows a typical example of two-dimensional trajectory of prickly ND when it was incubated with the cell in a serum free medium. The ND underwent random motion with rather large instantaneous velocity (Figure S5b) in the medium. When ND was anchored in the plasma membrane (orange rectangle in Figure S5a), its

motion became very restricted—the trajectory range was limited and the ND's instantaneous velocity dropped to below 0.5 μm/s (orange rectangle in Figure S5b). The fluctuation of caging diameter is consistent with the motion state, which dropped from above 5 μm in the medium to below 0.5 μm on the plasma membrane.

Once internalized, the NDs showed complex and varied motion behaviour, with both direct motion, confined motion and the mixed state can be observed (Figure S5 d, e, f). The trajectory of direct motion with large instant velocity and caging diameter, is consistent with the literature reports on particle travelling in cytoplasm, which was usually ascribed to NPs/vesicles travelling along cytoskeleton in the presence of motor protein[12,13].

The trajectory and velocity of ND underwent confined motion is quite similar to those anchoring on plasma membrane, but with relatively large caging diameter (above 0.5 μm, Figure S5 f III), probably due to the higher fluidity of cytoplasm than the plasma membrane. The trajectory shown in Figure S5 d II consist of a period of direct motion and two confined period at the start and end. The increase of caging diameter is consistent with the direct motion in the middle.

The significantly different behaviour of NDs anchored on plasma membrane and travel in cytoplasm is further illustrated in the distribution of velocity obtained by analysing multiple trajectories (Figure S5 g). The NDs anchored on plasma membrane have a much narrower distribution of velocity compare with those traveling in cytoplasm, consistent with the above analysis.

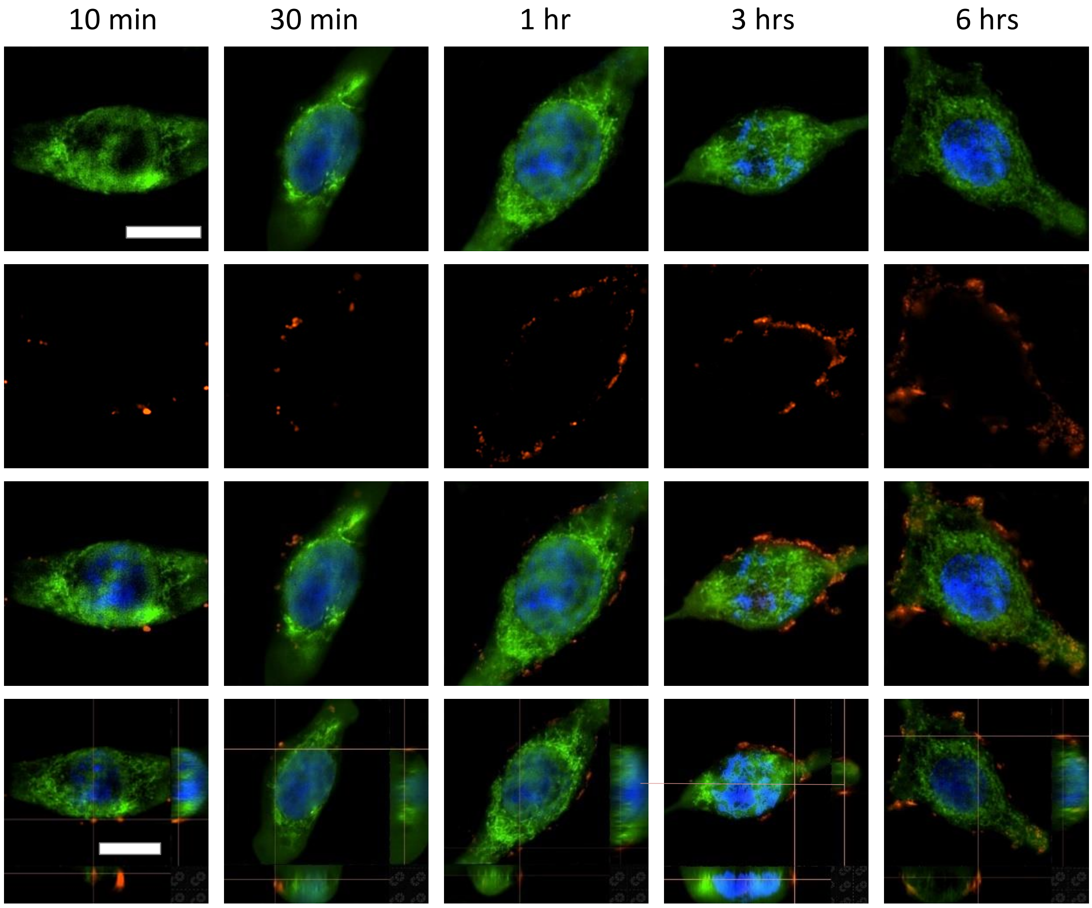

Figure S6. Typical N-SIM images of prickly NDs incubated for 10 min, 30 min, 1 hr., 3 hrs. and 6 hrs. at 4 ^{o}C, cross sectioned views indicate the NDs are mostly on the cell membrane. (blue: DAPI-nucleus, green: cell tracker-cytoplasm, red: ND. Scale bar: 20 μm, the images in the first three rows share the same scale bar and the images in the last row share the same scale bar)

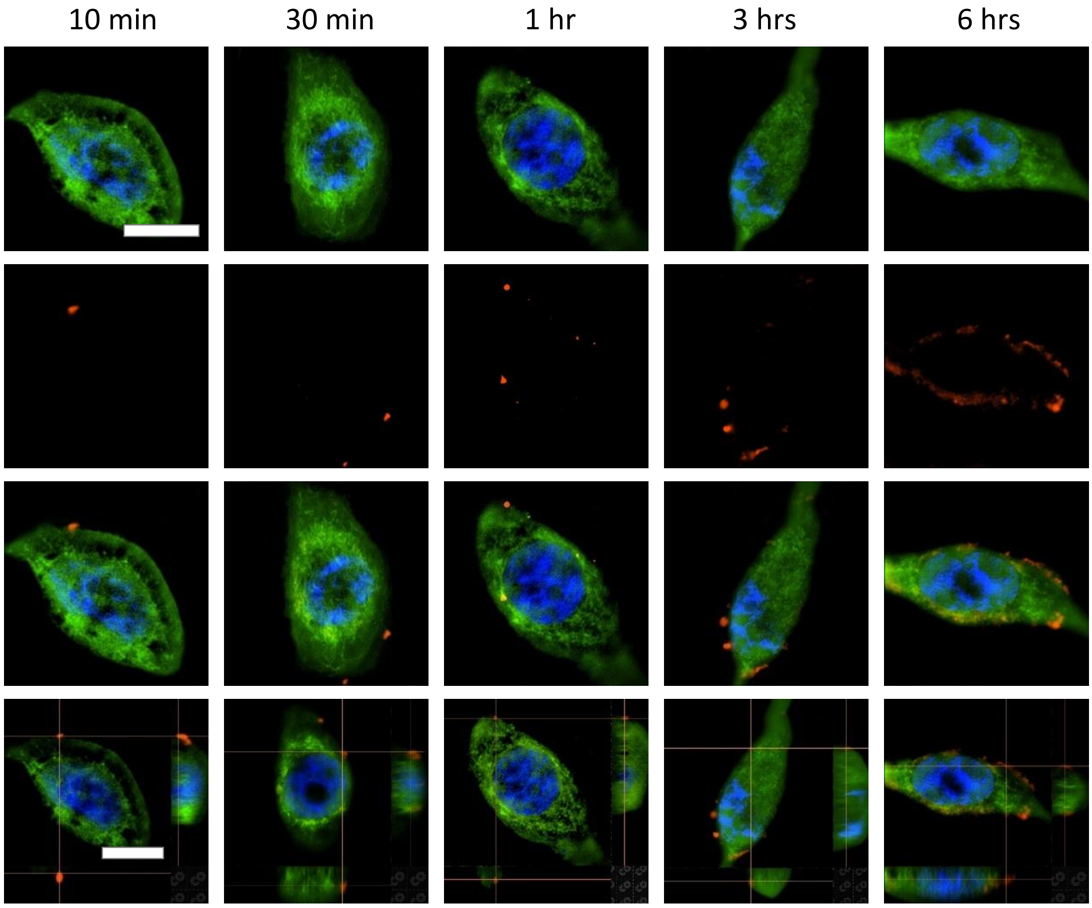

Figure S7. Typical N-SIM images of round NDs incubated for 10 min, 30 min, 1 hr., 3 hrs. and 6 hrs. at 4 ^{o}C, cross sectioned views indicate the NDs are mostly on the cell membrane. (blue: DAPI-nucleus, green: cell tracker-cytoplasm, red: ND. Scale bar: 20 µm, the images in the first three rows share the same scale bar and the images in the last row share the same scale bar)

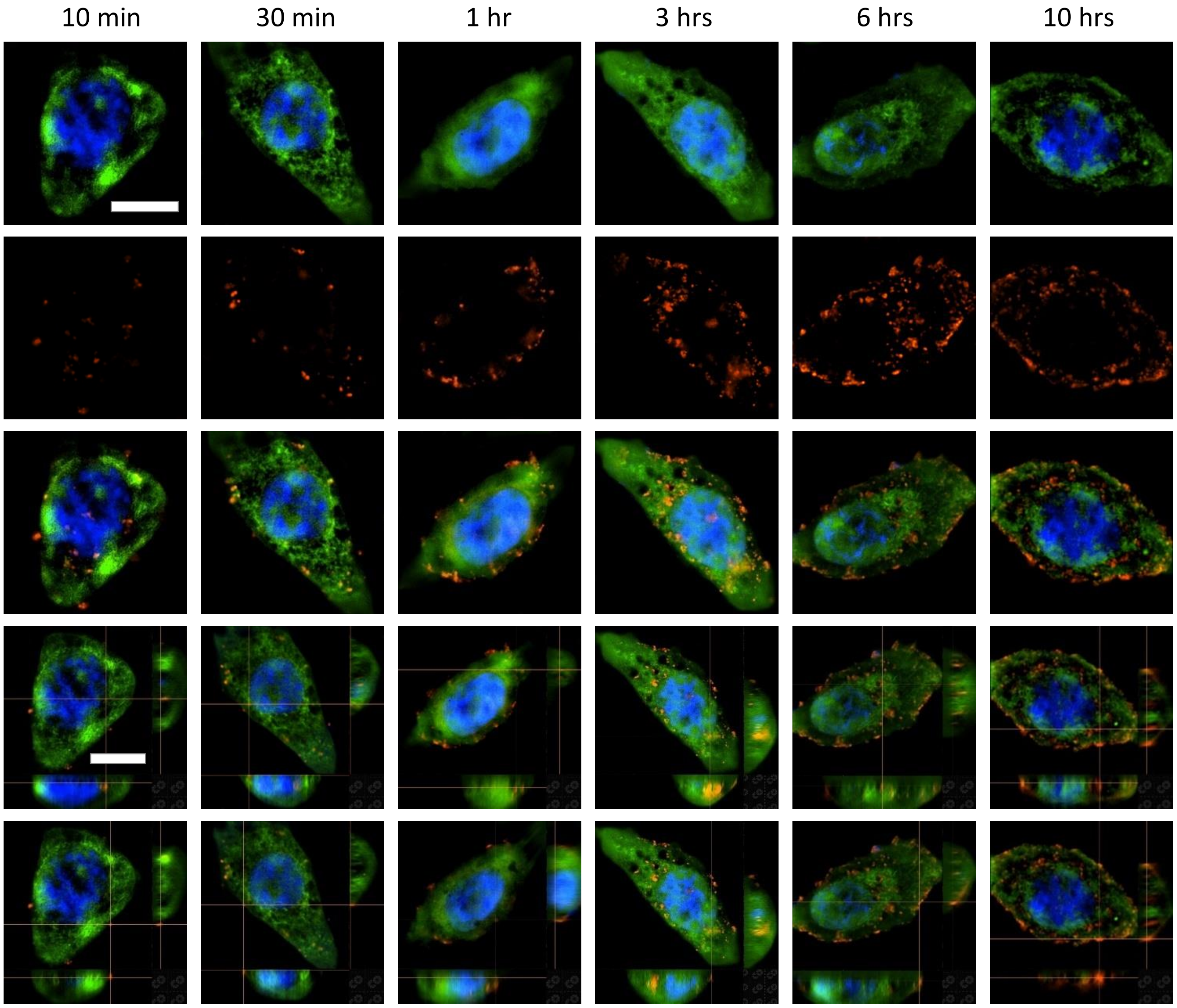

Figure S8. Typical N-SIM images of prickly NDs incubated for 10 min, 30 min, 1 hr., 3 hrs., 6 hrs. and 10 hrs. at 37 °C, cross sectioned views indicate the NDs are both inside the cell and on the cell membrane. (blue: DAPI-nucleus, green: cell tracker-cytoplasm, red: ND. Scale bar: 20 µm, the images in the first three rows share the same scale bar and the images in the last two rows share the same scale bar)

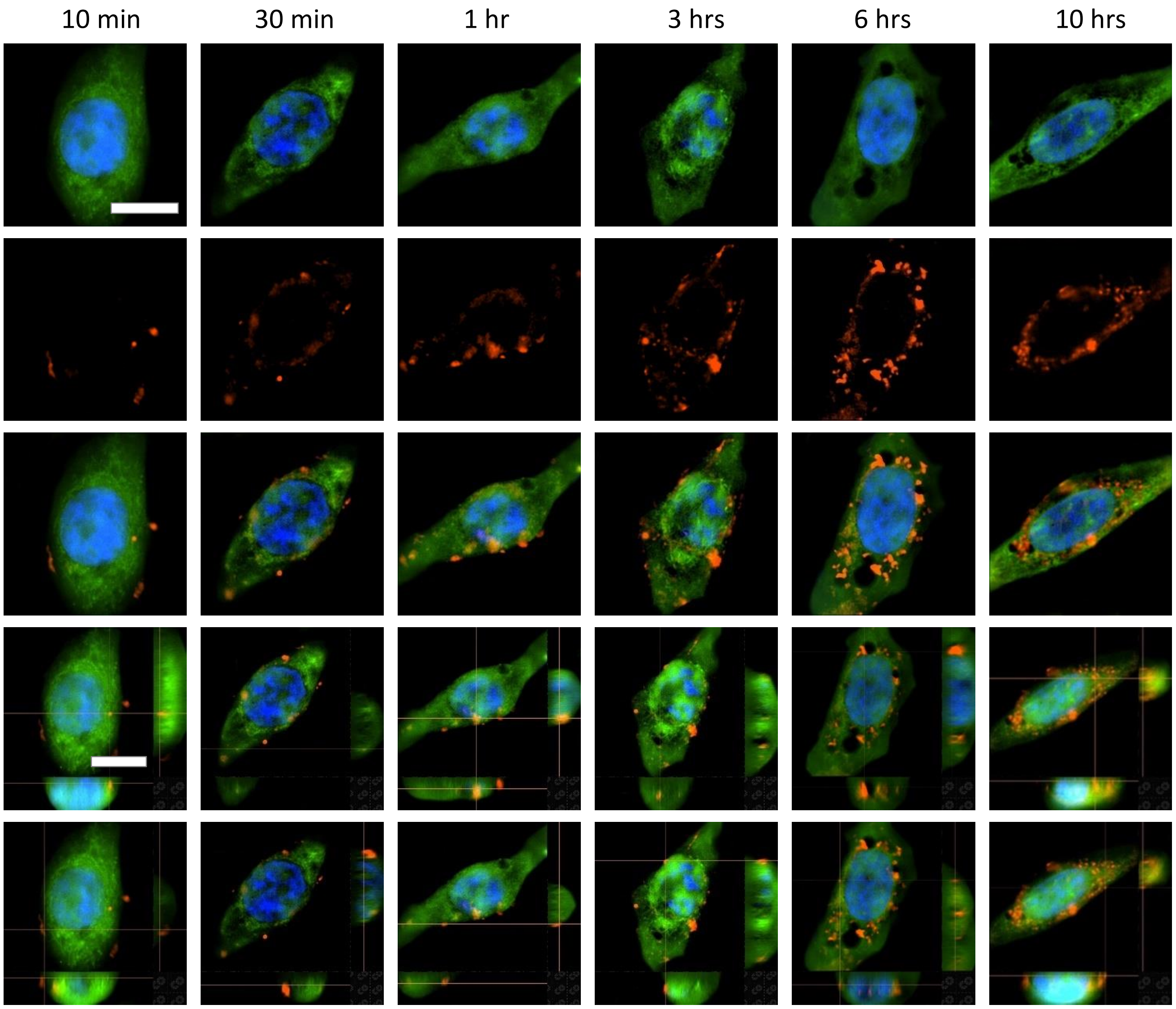

Figure S9. Typical N-SIM images of round NDs incubated for 10 min, 30 min, 1 hr., 3 hrs., 6 hrs. and 10 hrs. at 37 °C, cross sectioned views indicate the NDs are both inside the cell and on the cell membrane. (blue: DAPI-nucleus, green: cell tracker-cytoplasm, red: ND. Scale bar: 20 µm, the images in the first three rows share the same scale bar and the images in the last two rows share the same scale bar)

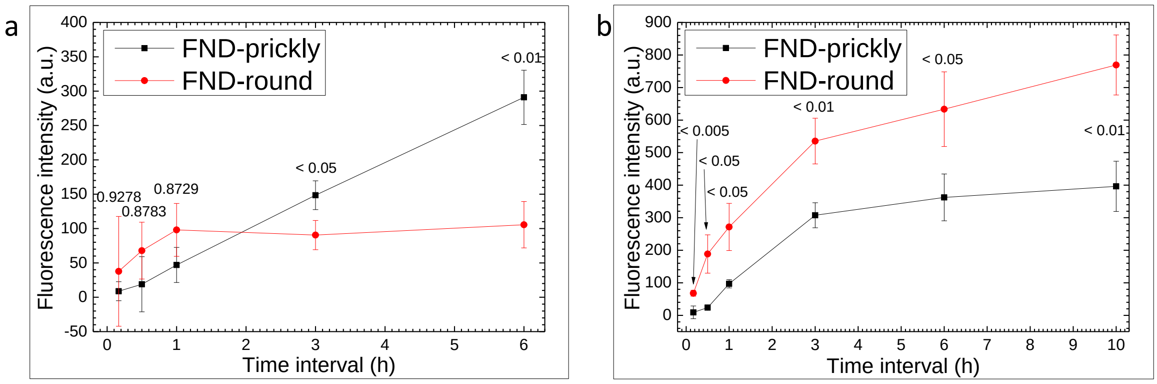

Figure S10. Quantitative fluorescence intensity of prickly and round NDs fed HeLa cells. (a) Fluorescence intensity of ND Anchored on the plasma membrane at 4 °C. (b) Fluorescence intensity of ND (both anchored and Internalized) at 37 °C, the amount of the internalized round shape NDs was ~2 times higher than that of the prickly shape NDs. (Error bar represents SD. Inserted numbers are p values for the comparison at each time point, $n = 4$.)

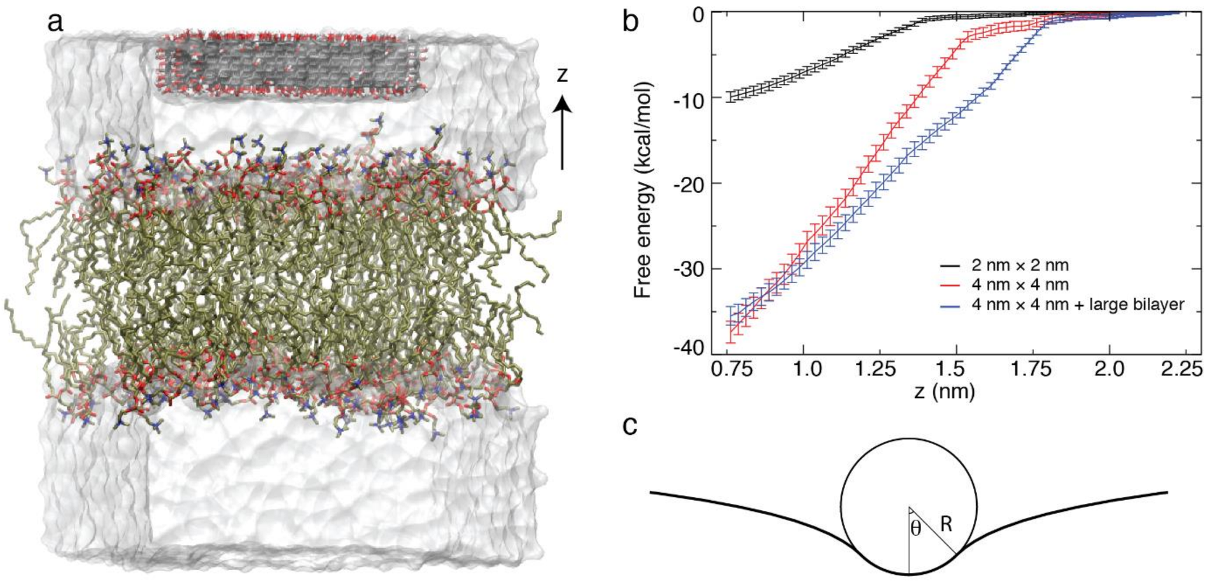

Figure S11. Calculation of k_{ad} via umbrella sampling and wrapping of a ND tip via continuum modeling. (a) MD simulation system used to determine the free energy profile of ND-membrane association. (b) The free energy profile of ND-membrane association. The ND slab is in contact with the membrane at d≈0.8nm. A free energy minimum of -9.93±0.60 kcal/mol, -37.39±1.27 kcal/mol and -35.48±1.08 kcal/mol was obtained for the 2nm x 2nm and 4nm x 4nm ND slabs and 4nm x 4nm ND slab with a large bilayer, respectively, which correspond to k_{ad} = -2.48±0.15 kcal/mol/nm^2, -2.33±0.08 and -2.22±0.07 kcal/mol/nm^2, respectively. Uncertainty in the results was computed using 1000 rounds of boot-strapping analysis with the GROMACS g_wham tool (using the trajectory method). (c) Schematics showing membrane wrapping of a ND tip by an infinite, tension-less membrane. θ stands for the wrapping angle and R is the radius of the ND tip.

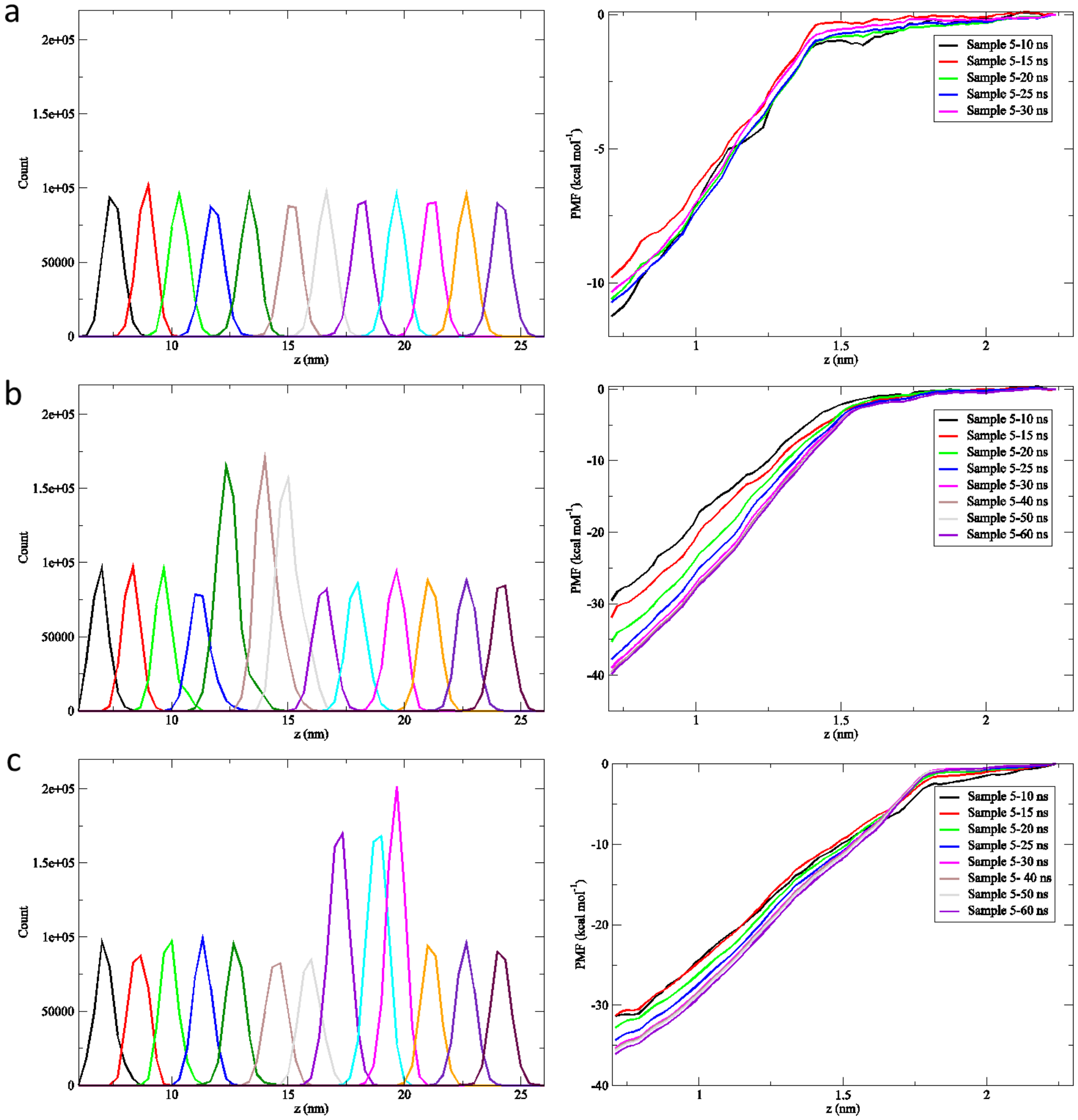

Figure S12 Number of counts in each umbrella window and the convergence of the potential of mean force in umbrella sampling of (Top) 2 nm × 2 nm ND slab system, (Middle) 4 nm × 4 nm ND slab system and (Bottom) 4 nm × 4 nm ND slab system with a large bilayer. The first 5ns of each umbrella window is considered as equilibration. Convergence is demonstrated by showing PMFs computed using an increasing amount of sampling time from each umbrella window (certain windows were extended to 60 ns as described in the Methods section).

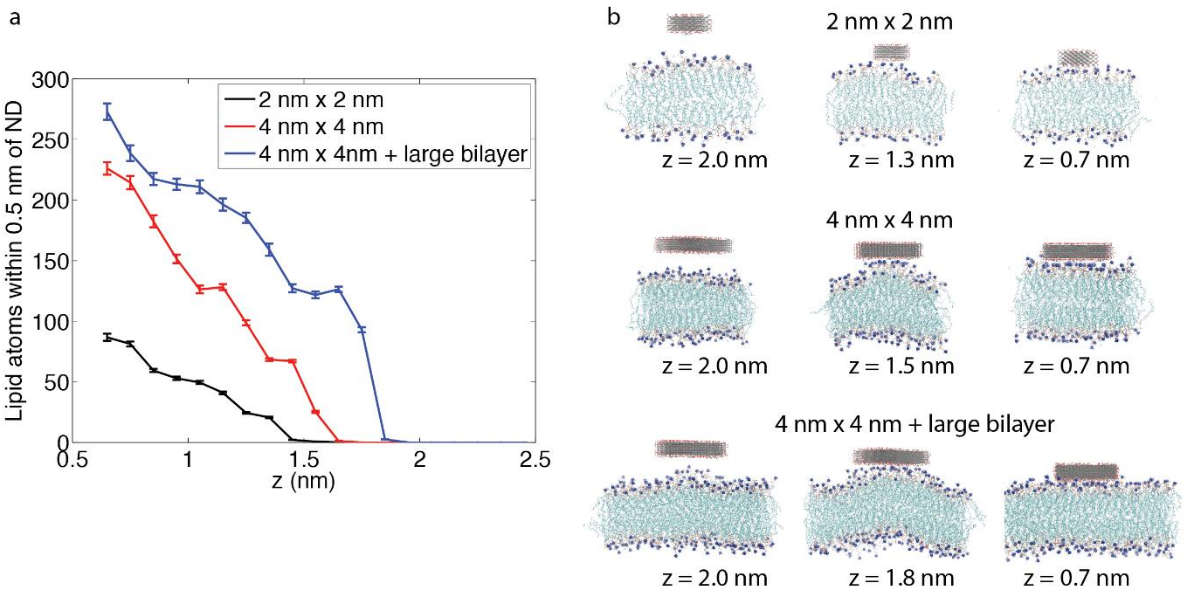

Figure S13. (a) The number of lipid atoms within 0.5 nm of the ND slabs. The calculation was performed using the umbrella sampling trajectories and subsequently binned to yield the average value at a given z. Standard errors were obtained as $S_i/(N_i/g_i)^{1/2}$, where S_i and N_i are the standard deviation and sampling number of bin i, respectively, while g_i is the statistical ineffciency of the umbrella window centered nearest bin i. (b) Snapshots of umbrella sampling trajectories for the three systems shown in (a).

References

1. Ge, Z., Li, Q. & Wang, Y. Free Energy Calculation of Nanodiamond-Membrane Association—The Effect of Shape and Surface Functionalization. *J. Chem. Theory Comput.* **10,** 2751–2758 (2014).
2. Hong, C., Tieleman, D. P. & Wang, Y. Microsecond Molecular Dynamics Simulations of Lipid Mixing. *Langmuir* **30,** 11993–12001 (2014).
3. Phillips, J. C. *et al.* Scalable molecular dynamics with NAMD. *J. Comput. Chem.* **26,** 1781–1802 (2005).
4. Vanommeslaeghe, K. *et al.* CHARMM general force field: A force field for drug-like molecules compatible with the CHARMM all-atom additive biological force fields. *J. Comput. Chem.* NA-NA (2009). doi:10.1002/jcc.21367
5. Klauda, J. B. *et al.* Update of the CHARMM All-Atom Additive Force Field for Lipids: Validation on Six Lipid Types. *J. Phys. Chem. B* **114,** 7830–7843 (2010).
6. Hans C. Andersen. RATTLE: A 'Velocity' version of the SHAKE algorithm for molecular dynamics calculations. (1983).
7. Miyamoto, S. & Kollman, P. A. SETTLE: an analytical version of the SHAKE and RATTLE algorithm for rigid water models. *J. Comput. Chem.* **13,** 952–962 (1992).
8. Darden, T., York, D. & Pedersen, L. Particle mesh Ewald: An N·log(N) method for Ewald sums in large systems. *J. Chem. Phys.* **98,** 10089 (1993).
9. Feller, S. E., Zhang, Y., Pastor, R. W. & Brooks, B. R. Constant pressure molecular dynamics simulation: The Langevin piston method. *J. Chem. Phys.* **103,** 4613 (1995).

10. Deserno, M. Elastic deformation of a fluid membrane upon colloid binding. *Phys. Rev. E* **69,** (2004).

11. Nofal, S., Becherer, U., Hof, D., Matti, U. & Rettig, J. Primed Vesicles Can Be Distinguished from Docked Vesicles by Analyzing Their Mobility. *J. Neurosci.* **27,** 1386–1395 (2007).

12. Zajac, A. L., Goldman, Y. E., Holzbaur, E. L. F. & Ostap, E. M. Local Cytoskeletal and Organelle Interactions Impact Molecular-Motor-Driven Early Endosomal Trafficking. *Curr. Biol.* **23,** 1173–1180 (2013).

13. Nan, X., Sims, P. A., Chen, P. & Xie, X. S. Observation of Individual Microtubule Motor Steps in Living Cells with Endocytosed Quantum Dots. *J. Phys. Chem. B* **109,** 24220–24224 (2005).
